# Compact optical fiber photoacoustic gas sensor with integrated multi-pass cell

**DOI:** 10.1016/j.pacs.2023.100524

**Published:** 2023-06-20

**Authors:** Enbo Fan, Haojie Liu, Chao Wang, Jun Ma, Bai-Ou Guan

**Affiliations:** a Guangdong Provincial Key Laboratory of Optical Fiber Sensing and Communications, Institute of Photonics Technology, Jinan University, Guangzhou 511443, China; b The Center for Smart Sensing System, Julong College, Shenzhen Technology University, Shenzhen 518118, China

**Keywords:** Fiber-optic sensor, Photoacoustic spectroscopy, Fabry-Perot cavity, Multipass cell, Acoustic wave

## Abstract

Optical fiber acoustic sensors with miniature size and high sensitivity are attractive to develop compact photoacoustic spectroscopy. Here, a compact photoacoustic gas sensor was demonstrated by utilizing a diaphragm-based fiber-optic Fabry-Perot cavity as both the acoustic sensor and the multipass cell. A nanoscale graphite film was used as the flexible diaphragm to increase the acoustic sensitivity of the Fabry-Perot cavity and the cavity inner surface was coated with highly-reflective Au film to form a multipass cell for amplification of the photoacoustic signal. With a laser power of 20 mW at 1532.8 nm, the sensor demonstrated a low detection limit of ∼ 50 ppb for C_2_H_2_ gas with an integration time of ∼ 100 s. The optical fiber photoacoustic gas sensor with a millimeter-scale diameter and ppb-level detection limit is promising for trace gas sensing in various areas including industrial process and environmental monitoring.

## 1 Introduction

Photoacoustic spectroscopy (PAS) is a sensitive gas measurement technique that relies on the detection of the acoustic waves generated from the thermal expansion and contraction of gas molecules absorbing intensity/wavelength modulated light [1], [2], [3]. By using laser sources such as diode lasers, interband cascade lasers (ICLs) and quantum cascade lasers (QCLs) to cover the spectral fingerprint of gas molecules ranging from visible to infrared band, diverse gas species can be measured in high sensitivity and specificity. Different from the direct absorption spectrum (DAS), PAS employed a microphone to detect the locally-generated photoacoustic (PA) waves, thereby avoiding the use of a photodetector which is expensive at the infrared band. Moreover, there is no need for a long gas cell to accumulate the gas absorption to obtain sufficient sensitivity [4], [5], which makes the PAS attractive to develop compact gas sensors for space-limited and remote sensing applications [6], [7].

Due to the large condense microphones with an element size of several millimeters to centimeters, the early PAS systems commonly employed relatively bulky gas cells to incorporate the microphones [8], [9]. Reducing the size of the microphone can allow the use of a compact gas cell but at the expense of the PAS sensitivity considering the tradeoff between the acoustic sensitivity and the element size of the microphone. Although PA signals can be amplified by boosting up the pump power to Watts level via an optical amplifier [10] or using an acoustic resonator to accumulate the acoustic energy [11], the high-power laser source adds the system cost and the additional acoustic resonant structure inevitably increases the sensor size again. In comparison, quartz-enhanced PAS employs a tiny tuning fork to detect the acoustic waves between its two prongs facilitating the miniaturization of the sensor and have shown excellent sensitivity leveraging the strong mechanical resonance of the tuning fork to amplify the PA signals [12], [13], [14], [15]. More recently, fiber-optic microphone with high sensitivity and miniature size have also drawn wide attentions for miniaturized PAS [16], [17], [18], [19], [20], [21]. Compared to the tuning fork, the fiber-optic microphone can not only reduce the gas sensor size but also enable remote sensing capability as no free-space light alignment is involved. Two widely used type of fiber-optic microphones for PAS are ferule-top cantilever and Fabry-Perot (F-P) cavity. They both work in an interferometric configuration and can detect extremely weak acoustic wave, which enables the detection of trace gas with the concentration in the parts per million (ppm) to parts per billion (ppb) level. In particular, the fiber-optic Fabry-Perot microphone employs a rigidly clamped circular membrane as the sensitive element, allowing the use of nanoscale thin elastic diaphragm to increase the acoustic pressure sensitivity rather than increasing its geometrical size [22]. A ppb-level detection limit has been achieved by the PAS sensor using a graphene film-based fiber-optic Fabry-Perot microphone with a diameter of only 2.5 mm [23], [24], [25]. However, in the previous fiber-optic F-P cavity PAS, a free-space laser light is collimated and delivered to the gas in the chamber to excite the photoacoustic waves while the fiber-optic F-P acoustic sensor is placed close to the pump light beam to pick up the acoustic waves. The whole setup still has a centimeter-scale size and more likes a benchtop device. The separate components including the collimator and the fiber-optic sensor also make the PAS system sensitive to mechanical vibrations.

**Fig. 1:**
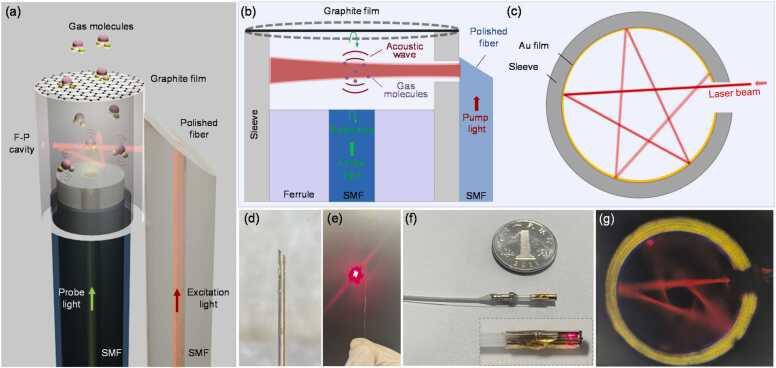
Schematic of (a) the PAS gas sensor based on fiber-optic F-P cavity with a flexible graphite film, (b) the excitation of PA signals from gas molecules and (c) the multipass beam travelling inside the cavity; (d) Photograph of the polished fiber tip with an titled angle of 45 degrees; (e) Visible light emitted from the fiber tip; (f) Photograph of the PAS gas sensor with the enlarged part shown in the inset; (g) Photograph of the multipass beam inside the cavity.

Here, a compact and portable PAS gas sensor based on a flexible graphite diaphragm-based fiber-optic F-P cavity as shown in Fig. 1(a) was demonstrated. The graphite film with a thickness of 100 nm was used as the flexible diaphragm to increase the acoustic sensitivity of the Fabry-Perot cavity without the expense of compactness. The F-P cavity simultaneously functioned as the PA gas cell, where an end-polished fiber tip assembled at the side wall of the cavity reflected the pump light into the gas cell for PA signal excitation. The inner surface of the F-P cavity was deposited with highly reflective Au film to form a multipass cell that amplifies the photoacoustic signal for 5 times. An end-polished fiber tip was assembled at the side wall of the Fabry-Perot cavity to deliver the pump light into the cavity, which avoided the collimation of free-space light. With a low pump power of only 20 mW at the wavelength of 1532.8 nm, the compact sensor demonstrated a low detection limit of ∼ 50 ppb for C_2_H_2_ gas under an integration time of ∼ 100 s.

## 2 Principle

The PA signals emitted from the excited gas molecules deflects the thin graphite diaphragm and subsequently modulates the F-P cavity length. The corresponding spectral shift of the F-P cavity reflection changes the intensity of the reflected probe light which is converted into electrical signals by a photodetector (PD). The PD output signal can be expressed as [23],

(1) $$S=S_{m}C_{cell}\alpha P,$$

where *S*_*m*_ is the pressure sensitivity of the acoustic sensor, *C*_*cell*_ is the PA cell constant reflecting the capability of converting the absorbed light energy into pressure and mainly depends on the cell geometry and acoustic frequency, *α* is the absorbance of the gas, and *P* is the power of the pump light. Based on Eq. (1), the amplitude of output signal from the PAS gas sensor is dependent on *S*_*m*_ and *P* if the absorption line of the target gas and the geometric parameters of the PA cell are specified. Therefore, the output signal of the gas sensor can be enhanced by increasing the sensitivity of the acoustic sensor and the pump light power.

To increase the acoustic pressure sensitivity of the fiber-optic F-P cavity, a thin elastic graphite diaphragm with a thickness of 100 nm was used to construct the fiber-optic F-P cavity since the pressure-induced deflection of an edge-fixed circular diaphragm is inversely proportional to the cube of its thickness [22]. The fabrication of the fiber-optic F-P cavity follows the same procedure as described previously [24]. The graphite film acts as the flexible mirror and forms the F-P cavity with the fiber tip facet as shown in Fig. 1(b). To reduce the sensor size, a ceramic ferrule with a smaller bore diameter of ∼ 1.2 mm compared with the previous study was used here.

To increase the pump power inside the gas cell, the inner surface of the ferrule was sputtering-coated with highly reflective Au film to form a compact multipass cell as shown in Fig. 1(c). For the Au film coating, the sputtering time and the current of the Au sputter were set at 600 s and 8 mA. The multipass cell has been used as an efficient manner to enhance the sensitivity for both DAS and PAS [26], [27], [28], [29], [30]. To deliver the pump light into the sleeve, a single mode fiber (SMF) tip was polished with a tilted angle of 45 degrees (Fig. 1(d-e)) and bonded onto the outer surface wall of the ferrule by ultraviolet (UV) curing gel (Fig. 1(f)). The polished fiber tip was prepared by firstly cleaving a SMF and then inserting it into the fiber holder of a polishing machine (Ultrapol, Ultra Tec). After tuning the tilted angle of the fiber holder to 45 degrees, the fiber tip was moved to a lapping sheet for polishing. The pump light from the distal pigtail was redirected by the polished fiber tip facet into the F-P cavity through its side slit and reflected back and forth as shown in Fig. 1(g). The gas molecules diffused into the multipass cell through the side slit of the ferrule and absorbed the pump light. The pump power *P* gradually decays during the back and forth travel due to the imperfect reflection (*R*) of the concave Au film. Based on Beer-Lambert law, the pump power *P* in the multipass cell can be expressed by [31],

(2) $${\left(\frac{P}{P_{0}}\right)}_{v}=\sum_{n=1}^{\infty }R^{n-1}e^{-\left(P_{g}CS_{i}\phi_{v}\right)nl}=\sum_{n=1}^{\infty }R^{n-1}e^{-\alpha_{v}nl}\approx \frac{e^{-\alpha_{v}l}}{1-Re^{-\alpha_{v}l}},$$

where *P*_*0*_ is the pump power incident into the cavity, *R* is the reflectivity of the concave Au film, *n* is the number of the reflection, *l* is the optical path length between two adjacent reflections, and *α*_*v*_= *P*_*g*_*CS*_*i*_*ϕ*_*v*_ is the absorption coefficient with *P*_*g*_, *C*, *S*_*i*_ and *ϕ*_*v*_ denoting the total gas pressure (atm), the gas concentration, the line strength of transition *i* (cm^−2^atm^−1^) and the line shape function (cm), respectively. The slight difference of the optical path length *l* between two reflections was neglected here for simplification. For trace gas detection, the optical transmission loss caused by the gas absorption can be ignored and then the equivalent pump power *P* can be increased for 1/(1-*R*) times based on Eq. (2). Therefore, a large value of *R* can significantly increase the pump power *P* inside the multipass cell and thus enhance the sensitivity of the PAS gas sensor.

## 3 Experimental section

### 3.1 Acoustic sensing

**Fig. 2:**
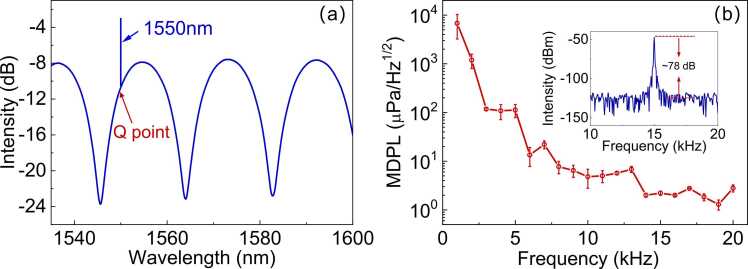
(a) Reflection spectrum of the PAS gas sensor with the arrow indicating the quadrature (Q) point for maximum acoustic sensitivity; (b) Frequency response of the sensor from 1 kHz to 20 kHz. Inset: frequency spectrum to acoustic wave at 15 kHz.

The reflection spectrum of the PAS gas sensor was measured with an optical spectrum analyzer (Yokogawa, AQ6370B) and a broadband source (Golight, SLED-1250–1650 nm) and is shown in Fig. 2(a). The fringe contrast of the reflection spectrum is larger than 15 dB, as a result of the high reflectivity (∼ 0.3) of the graphite film [22]. This large fringe contrast is beneficial to increase the acoustic sensitivity because of the large spectrum slope at the quadrature point during acoustic wave detection using a narrowband probe laser at the wavelength of 1550 nm.

For trace gas detection, the environmental disturbances such as air flow or temperature fluctuations may cause the spectral shift of the fiber-optic F-P cavity and thus lead to fluctuation in the output signals. The thermal effect caused by the absorbed portion of the pump power by the Au film may also affect the signal stability. To lock the quadrature point of the sensor to the probe laser at 1550 nm as indicated in Fig. 2(a), a low cost and efficient photothermal stabilization scheme instead of using an expensive tunable laser was employed [24]. The output power of a continuous wave (CW) 980 nm laser was servo-controlled with an electronic variable optical attenuator (EVOA) to heat and deflect the graphite film featuring a broadband absorption, which tuned the sensor reflection spectrum dynamically and locked the quadrature point to the wavelength of the 1550 nm probe laser via a PID algorithm. The frequency response of the stabilized sensor was then acquired by measuring the intensity change of the reflected probe light to acoustic waves generated from a loudspeaker driven by a signal generator. The pressure of the acoustic waves at the frequency from 1 kHz to 20 kHz was calibrated with a standard microphone (AWA5661, Aihua instrument). As the sensor was tested in a home-made anechoic box instead of a standard anechoic room, the interference of the acoustic waves from the sound source and those reflected from the microphone head and the wall of the anechoic box caused the spatial fluctuation of the pressure, which varied with the acoustic frequency [32]. Therefore, we measured three times to acquire the sensor frequency response by slightly varying the sensor position each time, with the averaged frequency response shown in Fig. 2(b). The minimum detection pressure level (MDPL) was obtained by using the electrical spectrum analyzer (R&S, FSV3000) to measure the signal to noise ratio of the sensor subjected to an acoustic pressure of ∼ 100 mPa, as shown in the inset of Fig. 2(b). At the frequency of 15 kHz, a SNR of ∼ 78 corresponds to a MDPL of ∼ 1.8 μPa/Hz^1/2^.

### 3.2 Experiment setup for photoacoustic gas sensing

**Fig. 3:**
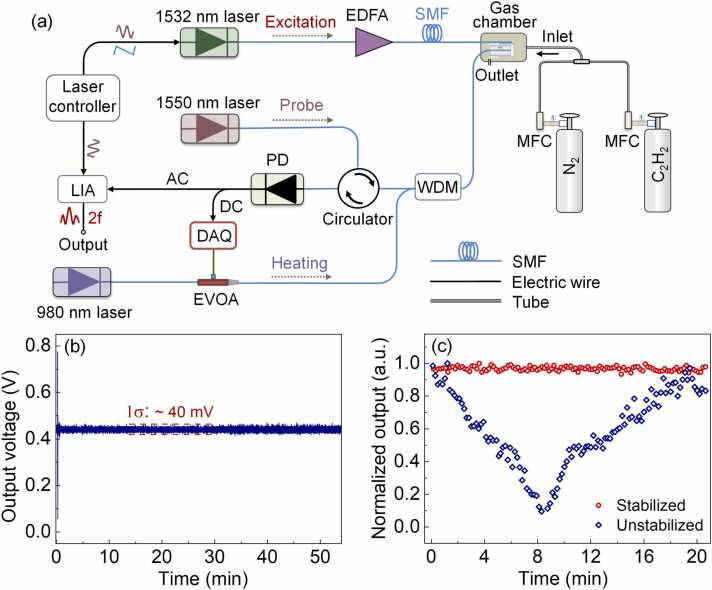
(a) Schematic of the experimental setup of the PAS system for C_2_H_2_ gas measurement; (b) Temporal feedback signal from the PD for a duration of 50 mins; (c) Comparison of the *2 f* signal from the sensor output before and after photothermal stabilization for the C_2_H_2_ gas with a concentration of 250 ppm.

The experimental setup of the PAS system for C_2_H_2_ gas measurement is shown in Fig. 3. The wavelength of the pump light from a distributed feedback (DFB) laser was slowly scanned over the gas absorption line at the 1532.8 nm and sinusoidally modulated by varying the temperature and driving current through the controllers (LTC56B, Thorlabs), respectively [33]. To obtain the maximum PA signal, we scanned the modulation frequency to search the optimal frequency which was ∼ 7 kHz. This frequency was lower than the supposed value of ∼ 9.5 kHz since the minimum MDPL located at 19 kHz as observed in Fig. 2(b). Different from the quartz tuning fork [12] or cantilever [14] featuring a sharp resonant peak, the sensor with a thin graphite film exhibited a relatively flat response in the range from 14 to 20 kHz, and thus the ripples of the sensor frequency response caused by the interference of the acoustic waves during the test might cause this discrepancy. The power of the pump light before reaching the gas sensor was amplified by an EDFA (C-BA-20, MRX-RRY Photonics) to 20 mW. To maximize the *2 f* signal, the modulation depth of the pump light wavelength was set to ∼ 2.2 times of the absorption linewidth [34]. The probe light at 1550 nm was delivered to the sensor after travelling through the circulator and wavelength division multiplexer (WDM). The reflected light from the sensor was converted into electrical signal by a photodetector (PD). The AC component of the electrical signal was measured by a lock-in amplifier (ziHF2, Zurich Instruments) with a time constant of 0.1 s and a filter slope of 18 dB/Oct, while the DC component was used as the feedback signal to photothermally stabilize the sensor by servo-controlling the EVOA (EVOA800A, Thorlabs) which rapidly tuned the heating power from 0 to 50 mW in a short time of ∼ 1 ms based on a PID algorithm-based LabVIEW program. The sensor was placed into a gas chamber which was continuously injected with the C_2_H_2_ gas through the inlet. The gas rates of the C_2_H_2_ and N_2_ gases were controlled by two mass flow controllers (MFCs) to regulate the C_2_H_2_ concentration. The feedback signal for the photothermal stabilization, i.e., the DC component of the output voltage from the PD, was recorded to monitor the temporal spectral shift of the sensor as plotted in Fig. 3(b) since the shift in the reflection spectrum wavelength would change the reflected intensity of the narrowband probe light received by the PD.

Once the stabilization program started, the variation of the feedback signal reduced from 0.7 V to 0.04 V and maintained at a relatively low amplitude over a duration of 50 min. To further verify the efficiency of the stabilization scheme for the trace gas measurement, the *2 f* signals from the stabilized sensor for the C_2_H_2_ gas with a concentration of 250 ppm at a pump power of 40 mW were compared with that from the sensor without stabilization. As shown in Fig. 3(c), the fluctuation of the *2 f* signals after the stabilization is significantly reduced to be less than 6%.

### 3.3 Gas sensing performance

**Fig. 4:**
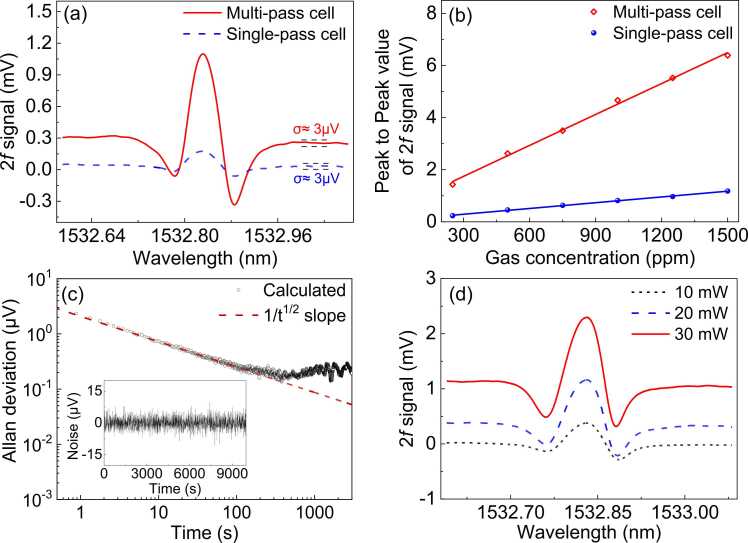
(a) Comparison of the second harmonic (*2 f*) signals from the PAS gas sensors with single-pass and multipass cells; (b) Peak-to-peak value of the *2 f* signal versus the gas concentration at the pump power of 20 mW; (c) Allan deviation plot for the sensor with multipass cell. Inset: temporal variation in the noise floor as the wavelength of the pump light was deviated from the gas absorption line; (d) *2 f* signal from the sensor with the multipass cell at different pump powers for a C_2_H_2_ concentration of 250 ppm.

The *2 f* signals from the sensor for the ferrule inner surface with and without Au coating at the C_2_H_2_ gas concentration of 250 ppm were compared in Fig. 4(a). For the case without Au coating, the gas cell can be considered as single pass due to the relatively low reflectivity of the ferrule surface. The peak-to-peak value of the *2 f* signal and the standard derivation (1*σ*) of the noise floor are estimated to be ∼ 0.3 mV and 3 μV, respectively. The calculated signal to noise ratio (SNR) is ∼ 100, corresponding to a detection limit of 2.5 ppm C_2_H_2_ gas. In comparison, the peak-to-peak value of the *2 f* signal from the sensor with Au-coated ferrule or the multipass cell is ∼ 1.4 mV, about five times enhanced compared to the sensor with a single-pass gas cell. At the pump power of 20 mW, the noise floor keeps nearly the same, which means that the gas detection limit is reduced to 0.5 ppm by the multipass cell. Based on Eq. (2), the reflectivity of the concave Au film on the inner surface of the ferrule is estimated to be ∼ 0.8, which is lower than that of a flat Au film and can be increased by using a specially-designed sleeve with a square bore to form the gas cell with flat inner surfaces. The sensor exhibited a linear response with the increasing gas concentration in the range from 250 ppm to 1500 ppm and the measured peak-to-peak values of the *2 f* signals are shown in Fig. 4(b). To examine the long-term stability and detection limit of the sensor, the pump light was tuned and fixed to the wavelength in absent of the gas absorption line. The *2 f* signals from the sensor were continuously recorded with a duration over 2.5 h for the Allan deviation analysis. As shown in Fig. 4(c), the deviation voltage reduced to ∼ 300 nV at the average time of 100 s, which corresponds to a minimum detection limit of ∼ 50 ppb according to the sensitivity curve as plotted in Fig. 4(b). The calculated deviation curve follows an exact *t*^*−1/2*^ trend as indicated by the solid fitting line, suggesting that the PA system is dominated by the white noise [35], owing to the high efficiency of the photothermal stabilization scheme.

**Fig. 5:**
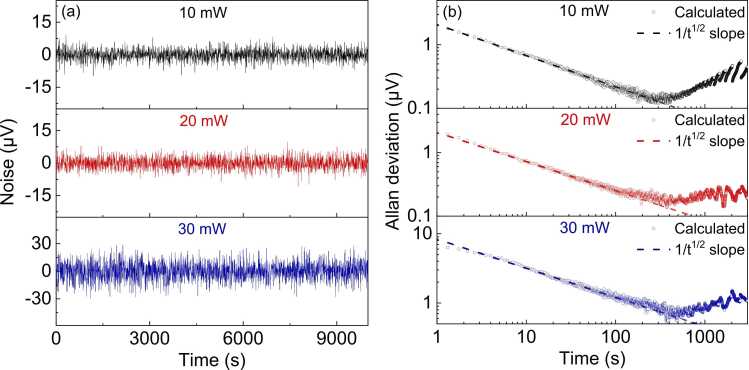
(a) Temporal variation of the noise voltages for the PAS gas sensor at different pump powers; (b) Allan deviation plots of the measured noise in (a).

To investigate the sensor performance at different pump powers, the operation current of the EDFA was tuned to change the pump power at 1532.8 nm for PA gas sensing. As shown in Fig. 4(d), the peak-to-peak value of the *2 f* signal increases with the pump power, indicating that the sensitivity of the PAS sensor can be further improved by increasing the pump power. However, the noise floor increased substantially once the heating power was raised up to 30 mW and caused a reduction in the SNR. The measured noises for a duration of over 2.5 h at different pump powers after removing the average voltage are shown in Fig. 5(a) and the corresponding Allan deviation curves are plotted in Fig. 5(b). The increased noise floor might be partly attributed to the increased background noise caused by the light absorption of the Au film. On the other hand, it was found that the stability of the sensor began to degrade as the pump power increased to more than 20 mW. This thermal effect from the Au film absorption can hardly be compensated by the photothermal stabilization program, which explains the rapid increase of the noise floor as the pump power increases from 20 mW to 30 mW. It is expected that this noise can be significantly alleviated through the reduction in the absorption of the Au film by improving the deposition quality using a high-vacuum magnetron sputtering in the future. It can be also observed in Fig. 4(d) that the signal bias voltage increases with the pump power, which might be caused by the absorption of the diffracted pump light inside the cavity by the graphite film. If the peak-to-peak of the 2 f signal is acquired for the gas measurement, the bias voltage will not affect the measured result. However, if the pump wavelength is fixed at the absorption line of the gas, this bias will offset the output voltage from zero when there is no gas sample. As a result, after the optimum pump power is selected, the bias voltage needs to be subtracted from the measured signal to retrieve the true gas concentration.

**Fig. 6:**
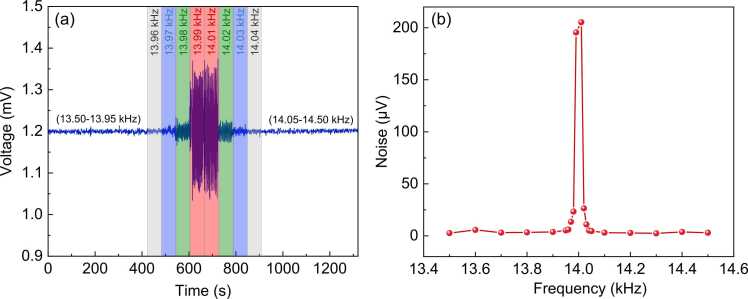
(a) Measured 2 f signals from the sensor subjected to applied external acoustic waves at different frequencies when the pump laser wavelength was fixed to the gas absorption line; (b) The noise voltage (1σ) as a function of the acoustic frequency.

As shown in Fig. 2(b), the sensor is also sensitive to the acoustic waves in the frequency range from 14 kHz to 20 kHz considering its relatively broad bandwidth. To study the effect of the environmental noise to the gas sensing performance of the system, we applied a quite strong acoustic waves (∼ 100 mPa) at different frequencies to the sensor by using the loudspeaker and simultaneously recorded its output voltages to the 250 ppm C_2_H_2_ gas after fixing the pump laser wavelength to the gas absorption line. As shown in Fig. 6, the output voltage and noise floor (1σ) maintain almost unchanged except at the frequency close to 14 kHz, which is the 2 f frequency selected for the gas measurement. The noise floor starts to increase significantly when the acoustic frequency approaches the frequency in the range from 13.97 kHz or 14.03 kHz, which is ∼ 30 Hz away from the 2 f frequency. Considering that the PA signal is generated inside the sensor inner cavity, it is possible to reduce the potential environmental noise located nearby the 2 f frequency by using a specially-designed tube encapsulated with porous sound-isolation materials that allow the gas diffusion into the cavity, which will be investigated in the future for practical applications.

## 4 Conclusion

In summary, a diaphragm-based fiber-optic Fabry-Perot cavity was employed as both the acoustic sensor and the multipass cell to build a compact photoacoustic gas sensor. By using a flexible graphite film with a thickness of 100 nm to increase the acoustic sensitivity and simultaneously an Au-coated multipass cell to amplify the PA signal, the sensor demonstrated a low detection limit of ∼ 50 ppb for C_2_H_2_ gas at an integration time of ∼ 100 s by using a low pump power of only 20 mW. In combination with the photothermal stabilization scheme that used a low-cost 980 nm laser, the immunity to environmental disturbance of the PAS system was also greatly enhanced. As a large reflectivity *R* of the concave Au film on the inner surface of the ferrule can significantly increase the pump power *P* inside the gas cell to enhance the sensitivity of the PAS gas sensor, improving the Au coating quality or using dielectric coating with high reflectivity and low absorption can be adopted despite the light diffraction by the concave film may ultimately limit the reflectivity *R*. With compact size and all-fiber light delivery, the optical fiber PAS gas sensor is promising for narrow space and remote sensing of trace gas in applications such as gas leakage detection, industrial process control and environmental monitoring.

## Declaration of Competing Interest

The authors declare that they have no known competing financial interests or personal relationships that could have appeared to influence the work reported in this paper.

## Data Availability

Data will be made available on request.
